# Probe my Pathway (PmP): a portal to explore the chemical coverage of the human Reactome

**DOI:** 10.1093/database/baae116

**Published:** 2024-12-03

**Authors:** Haejin Angela Kwak, Lihua Liu, Matthieu Schapira

**Affiliations:** Structural Genomics Consortium, University of Toronto, 101 College Street, MaRS South Tower, Suite 700, Toronto, Ontario M5G 1L7, Canada; Department of Pharmacology and Toxicology, University of Toronto, 1 King’s College Circle, Toronto, Ontario M5S 1A8, Canada; Structural Genomics Consortium, University of Toronto, 101 College Street, MaRS South Tower, Suite 700, Toronto, Ontario M5G 1L7, Canada; Structural Genomics Consortium, University of Toronto, 101 College Street, MaRS South Tower, Suite 700, Toronto, Ontario M5G 1L7, Canada; Department of Pharmacology and Toxicology, University of Toronto, 1 King’s College Circle, Toronto, Ontario M5S 1A8, Canada

## Abstract

Deciphering pathway–phenotype associations is critical for a system-wide understanding of cells and the chemistry of life. An approach to reach this goal is to systematically modulate pathways pharmacologically. The targeted and controlled regulation of an increasing number of proteins is becoming possible, thanks to the growing list of chemical probes and chemogenomic compounds available to cell biologists, but no resource is available that directly maps these chemical tools on cellular pathways. To fill this gap, we developed Probe my Pathway (PmP), a database where high-quality chemical probes and well-characterized sets of chemogenomic compounds are mapped on all the human pathways of the Reactome database. The web interface allows users to browse the data via icicle charts or search the data for compounds, proteins, or pathways. Chemists can rapidly find pathways with low chemical coverage or explore the structural chemistry of ligands targeting specific cellular machineries. Cell biologists can look for chemical probes targeting different proteins in the same pathway or find which pathways are targeted by chemical probes of interest. PmP is updated annually and will grow with the expanding chemical tool kit produced by Target 2035 and other efforts.

**Database URL:** https://apps.thesgc.org/pmp/

## Introduction

Chemical tools, such as chemical probes and chemogenomic compounds, are drug-like small molecules that modulate the activity of target proteins with a narrow and well-defined selectivity profile [1, 2]. These target proteins participate in signaling pathways or cellular machineries, the modulation of which determines cell function and cell fate. This clearly delineates a cause-and-effect path from chemical tools to proteins to cellular pathways to phenotype and underlines the value of chemical probes and chemogenomic compounds to explore and decipher human biology. Resources such as Chemical Probes Portal [3], Pharos [4], Probes and Drugs [5], and ChemBioPort [6] are available for matching chemical tools with target proteins. The Reactome [7] and KEGG [8] databases link proteins and chemicals, such as endogenous ligands or drugs to cellular pathways, while the iFragMent database [9] predicts the cellular pathways of a chemical compound of interest. To the best of our knowledge, no resource exists to map chemical probes or chemogenomics compounds onto human biological pathways. Yet, this information is highly valuable for medicinal chemists to highlight underexplored pathways, to understand the pharmacology of chemical tools, to reveal novel pathways of interest for drug discovery, and to ultimately link pathway to phenotype.

Having recognized the value of this information, we have conducted a comprehensive analysis of the coverage of human biological pathways by chemical tools [10]. In tandem, here we present Probe my Pathway (PmP), an online tool available at https://apps.thesgc.org/pmp/ to map high-quality chemical probes and chemogenomic compounds from well-characterized chemogenomic sets onto pathways present in the Reactome database. PmP can be used by chemists to identify potential targets of poorly explored or clinically interesting pathways and by cell biologists looking for chemical tools to modulate their pathways of interest. We provide an example illustrating each type of application.

### Data collection

The chemical probes were sourced from Chemical Probes Portal [3], Boehringer Ingelheim’s opnMe [11], and expert-vetted Donated Chemical Probes from the Structural Genomics Consortium (SGC) [12]. For probes compiled from the Chemical Probes Portal, only the ones given an in-cell rating of three or higher were included. The chemogenomic compounds consist of kinase and GPCR ligands from the kinase chemogenomic set (KCGS) V2.0 [13] released by the SGC and the EUbOPEN chemogenomic set. Human biological pathways and human proteins that belong in each pathway were extracted from the Reactome knowledgebase [7]. Upon completing data collection, compounds were mapped onto the human Reactome pathways using the Uniprot ID of the target proteins. The database currently contains 554 chemical probes, 484 chemogenomic compounds, 11 175 proteins, and 2673 pathways and is updated annually. Importantly, PmP is only populated with high-quality chemical tools, as opposed to poorly characterized or promiscuous compounds that all too often pollute the scientific literature [14, 15].

## Web interface

**Figure 1.**
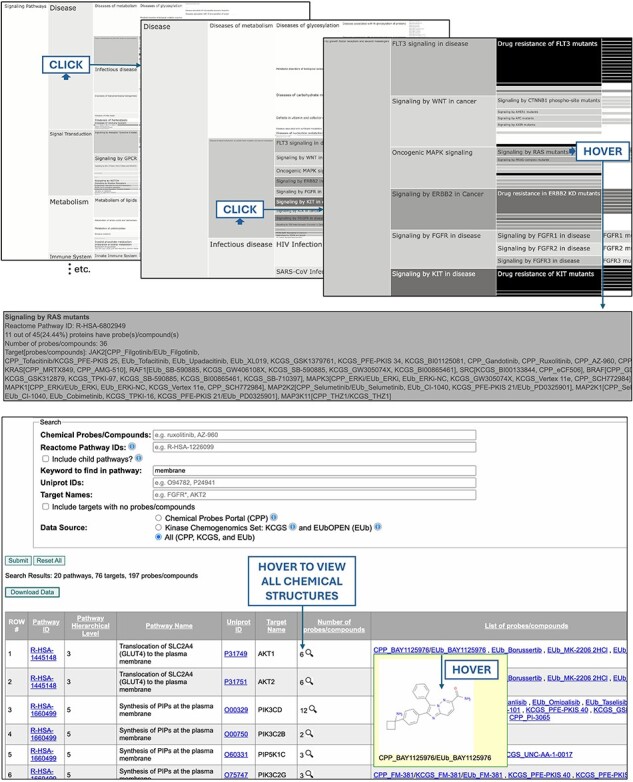
The PmP database can be browsed with interactive icicle charts, reflecting the mapping of chemical tools onto Reactome pathways (top), and a search engine returns the chemical coverage of pathways for any combination of text-based queries as a downloadable table (bottom).

Users can either browse or search the database. To browse the database, icicle charts for chemical probes, chemogenomic compounds, and the two compound types combined (“chemical tools”) are provided (Fig. 1, top). The architecture of the icicle charts reflects the hierarchical relationship between pathways (ex: “Signalling by receptor tyrosine kinases” is a child pathway of “Signal transduction”) and the shade-coding reflects the extent of the coverage of each pathway by chemical tools (dark: high coverage; light: low coverage). Hovering over a pathway in the icicle charts provides information on the number of proteins in the pathway, the percentage of proteins targeted by chemical tools, and the list of proteins with their respective chemical modulators. The icicle charts are interactive, allowing users to click and zoom in and out on pathways.

Users can also search the database by chemical tool, protein, pathway, or any combination thereof (Fig. 1, bottom). Cell biologists can choose to display all target proteins and their respective chemical probes for pathways matching a given keyword, such as “membrane” or “KRAS.” Chemists can search for pathways with limited chemical coverage, where new compounds would have a strong impact, or explore the chemical structures of compounds involved in a pathway of interest (ex: “signal transduction” or “cytokine signaling in immune system”). Users can include or omit child pathways as well as target proteins with no compound in the search output. The output is a downloadable table of pathways, proteins, and their chemical tools.

## Application

### For chemists

Pathways with poor chemical coverage can easily be identified from the shade-coding of the icicle charts. For instance, under the “disease” top-level pathway, the “disease of metabolism” child pathway is distinctly lacking coverage by chemical tools (Fig. 1, top). A chemist interested in metabolic disorders can search the corresponding Reactome pathway ID, R-HSA-5668914, with the “Include targets with no probes/compounds” checkbox checked, to get a full list of all corresponding proteins to see that IDH1 is the only protein of the 253 that is targeted by a chemical tool. They could then zoom-in further onto child pathways to select a novel target for chemical targeting. Conversely, a medicinal chemist may be interested in browsing the chemical structures associated with a pathway with high chemical coverage to gain insights into the associated structural chemistry, if any.

### For cell biologists

**Figure 2.**
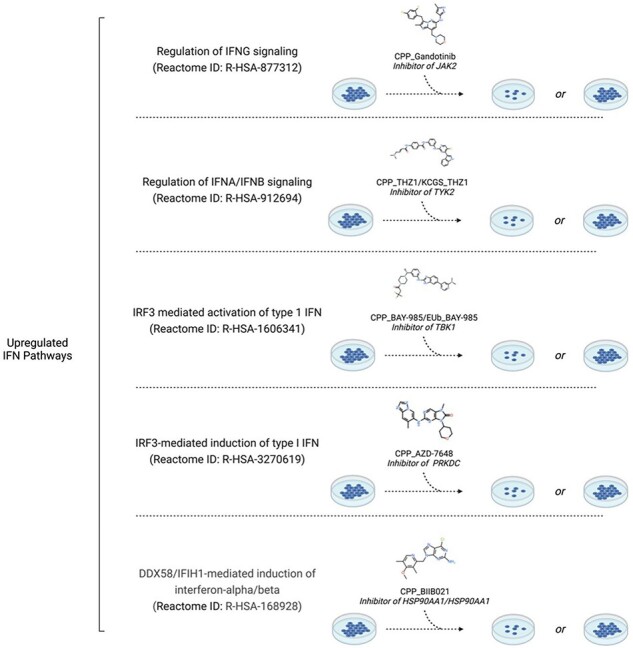
Cell biologists can use PmP, for example, to find chemical tools targeting distinct IFN pathways to dissect the mechanism of action of MS023, a Type I arginine methyltransferase inhibitor whose antitumor activity against triple-negative breast cancer depends on the activation status of IFN signaling. Created in BioRender. Szewczyk, M. (2023) https://BioRender.com/n99d265.

Cell biologists can use PmP to select chemical tools most appropriate to modulate a pathway of choice. For example, MS023 is an inhibitor of type 1 protein arginine methyltransferases. The activation of interferon (IFN) signaling before and after MS023 treatment determines antitumor growth activity in triple negative breast cancer (TNBC) [16]. To investigate which of the multiple IFN signaling pathways determine response to MS023 treatment, PmP can be used to identify chemical probes that inhibit each distinct IFN signaling pathway (Fig. 2). For instance, gandotinib is an inhibitor of JAK2 targeting the IFN-γ signaling pathway, the TYK2 inhibitor THZ1 targets IFN-α/IFN-β signaling, while the TBK1 and PRKDC inhibitors BAY-985 and AZD-7648, respectively, can be used to target IRF3-mediated modulation of Type I IFN. Once a probe for each pathway is identified, TNBC cell lines can be pretreated with each probe and treated with MS023 to observe whether inhibition of a specific pathway leads to the loss or maintenance of response (Fig. 2). PmP can also be used to easily check whether a protein targeted by a chemical tool is found in other pathways, which could obscure the mechanistic interpretation of phenotypic responses.

## Conclusion

Chemical probes can be used to reveal target–phenotype association, but a mechanistic and system-wide understanding of human biology can only be achieved by the controlled and targeted regulation of biological pathways. As the compendium of chemical tools available to inhibit or activate human proteins grows under the impulse of the Target 2035 initiative [17, 18], so does the chemical tool kit to finely regulate signaling pathways. We believe that PmP is a unique portal to assess the chemical coverage of the human Reactome and a valuable resource to guide chemistry efforts toward poorly characterized areas of human biology and to assist cell biologists in selecting the best possible compounds for their experiments.

## Data Availability

PmP is publicly available at https://apps.thesgc.org/pmp/. Data can be downloaded directly from the portal.
